# ZnGa_2−*x*_Al_*x*_O_4_ (*x* = 0 ≤ 2) spinel for persistent light emission and HER/OER bi-functional catalysis†

**DOI:** 10.1039/d3ra05017c

**Published:** 2023-10-24

**Authors:** Reshmi Thekke Parayil, Santosh K. Gupta, Manodip Pal, Arnab Dutta, Deepak Tyagi, Kathi Sudarshan, Manoj Mohapatra

**Affiliations:** a Radiochemistry Division, Bhabha Atomic Research Centre Trombay Mumbai 400085 India santoshg@barc.gov.in; b Homi Bhabha National Institute Anushaktinagar Mumbai 400094 India; c Chemistry Department, Indian Institute of Technology Bombay Powai Mumbai 400076 India arnab.dutta@iitb.ac.in; d Interdisciplinary Program in Climate Studies, Indian Institute of Technology Bombay Powai Mumbai 400076 India; e Chemistry Division, Bhabha Atomic Research Centre Trombay Mumbai 400085 India

## Abstract

Spinel materials have demonstrated diverse applications in various fields, especially in the energy sector. Since the pure spinel structure has the limitations of poor inherent activity and low conductivity, defect engineering through octahedral B-site modulation is expected to enhance various properties. Here in this work, we have synthesized ZnGa_2−*x*_Al_*x*_O_4_ (*x* = 0 ≤ 2) spinel and moved from one terminal (ZnGa_2_O_4_) to the other (ZnAl_2_O_4_) by varying the Ga/Al ratio using solvent-free solid-state reaction. Dopant and rare earth element-free (RE) ZnGa_2_O_4_ spinel showed excellent blue luminescence with photoluminescent quantum yields (PLQY) of 13% while exhibiting persistent light emission close to 60 min. The Al^3+^ incorporation at Ga^3+^ site doesn't yield any improvement in persistent luminescence lifetime owing to quenching of shallow traps as suggested by thermoluminescence (TL) studies. Moreover our materials have demonstrated bifunctional electrocatalytic activity towards both oxygen evolution (OER) and hydrogen evolution reaction (HER) which has never been reported for ZnGa_2−*x*_Al_*x*_O_4_. X-ray photoelectron spectroscopy (XPS) and positron annihilation lifetime spectroscopy (PALS) suggested that mixed Al/Ga-containing spinels possessed enhanced oxygen vacancies/defects. This makes them better electrocatalyst towards OER and HER compare to ZnGa_2_O_4_ and ZnAl_2_O_4_. The ZnGa_1.75_Al_0.25_O_4_ composition by virtue of enhanced oxygen vacancies and less charge transfer resistance (47.3 ohms) demonstrated best electrocatalytic activity for OER compared to the other synthesized catalysts at the same applied potential (1.6 V). On the other hand, the ZnGa_1_Al_1_O_4_ composition demonstrated excellent faradaic efficiency of ∼ 90% towards HER. From this work we can achieve multifunctional applications towards optoelectronics and electrocatalysis just by modulating Al/Ga ratio in ZnGa_2−*x*_Al_*x*_O_4_.

## Abstract 2

The unique PL and afterglow properties show the potential of ZnGa_2_O_4_ for energy and solid-state lighting applications. Solid solution of ZnGa_2_O_4_ and ZnAl_2_O_4_ showed promise as an efficient bifunctional electrocatalyst for both HER and OER.

## Graphical abstract

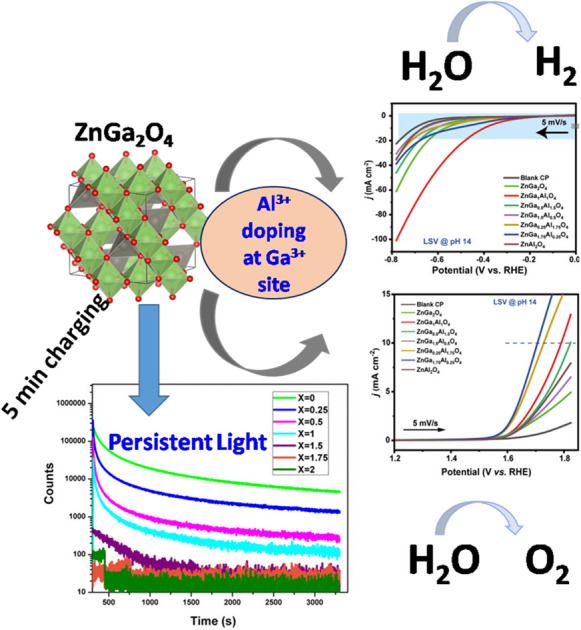

## 1. Introduction

A multifunctional material can be a boon towards a sustainable and developing economy, particularly in the area of energy, health, and environment.^1,2^ The two most important priorities in the area of the energy sector are designing an (a) inexpensive and efficient persistent luminescent materials and (b) efficient electrocatalysts for green hydrogen generation.^3,4^ Moreover, such a development will be pragmatic if it can be achieved without any involvement of rare earth elements and noble metals (*e.g.*, Pt, Ir or Ru).

For the development of a carbon-neutral society, the use of hydrogen as a fuel is inevitable since it produces benign water vapor and heat as byproducts. Currently, majority of the hydrogen production is executed by following steam methane reformation and coal gasification. However, these processes produce a copious amount of undesirable CO_2_.^5,6^ In this context, green hydrogen production *via* electrolysis emerges as a reliable carbon footprint-free technology.^7^ The electrochemical process typically has a high activation energy barrier for the reaction, where the presence of an electrocatalyst becomes essential to enhance the overall electrocatalytic energy efficiency.

The water-splitting process involves two reactions: hydrogen evolution reaction (HER) at the cathode and oxygen evolution reaction (OER) at the anode. The relative energies of chemical adsorption of H-atoms on the electrode surface and their subsequent electrochemical or chemical desorption regulates the electrocatalytic HER.^8^ The OER reaction is kinetically a sluggish reaction that proceeds *via* 4-electron transfer.^9^ Pt/C and Ru/IrO_2_ are considered as the benchmark electrocatalytic material for HER and OER, respectively.^10^ As large-scale productions of these materials are not suitable owing to their expensive and rare nature, the scientific community is actively exploring other abundant and low-cost electrocatalysts. Spinel oxides have been a popular choice in this regard due to their stable structure, adjustable valency, environmental safety and low cost.^11^ Spinel is an important class of compounds having the formula AB_2_O_4_ where A and B are divalent and trivalent cations, respectively. The spinel can be either normal or inverse depending upon the arrangement of A or B cations in the tetrahedral or octahedral site. For a normal spinel structure, A^2+^ occupies the tetrahedral whereas B^3+^ occupies the octahedral site. In the inverse spinel structure half of the B^3+^ ions occupy the tetrahedral and half of them occupy octahedral site, and A^2+^ ions cover the octahedral site.^12^ But pure spinel is not a good candidate for catalysis owing to their many limitations, such as poor inherent activity and low conductivity.^13^ So the defect engineering plays a crucial role in modulating the catalytic activity of spinels.

Defect engineering is one of the booming areas and a promising field for material chemists to synthesize various materials with enhanced catalytic, electrical, thermal, magnetic, and optical properties.^14–17^ This area is acquiring more importance recently due to its many applications in the various technological fields in the area of phosphors,^18^ scintillator,^19^ batteries,^20^ energy storage,^21^ electrocatalysis,^16,17,22^ thermoelectricity,^23^ and photocatalysis.^24^ The defects may be cationic vacancy, anionic vacancy (oxygen vacancy), and vacancy associates.^25^ There are many strategies employed for defect creation, which include aliovalent doping, annealing in a reduced atmosphere, electrochemical reduction, plasma irradiation, and amorphization.^26,27^

Persistent luminescence is a phenomenon in which the luminescence persists for a longer time, even after the stoppage of excitation. This phenomenon has many applications in fields like bioimaging, cancer therapy, night vision materials, and anti-counterfeiting.^28^ Traps play a vital role in persistent luminescence since these are the centers for storing the excitation energy, which is then transferred to emission center to initiate emission.^29^ The persistent intensity, as well as the emission time, depends on the trap structure and trap depth that is directly linked to lattice defects.^30,31^ This unique feature of the spinels was utilized in designing bright and long duration persistent light emitting materials (PLEMs).^19,28,32^ The tactical addition of dopant ions is primarily applied to invoke significant lattice distortion and strain, which affects the light emitting properties of the phosphor. Furthermore, there are lots of studies on the afterglow materials which employ rare earth (RE) ions that are not only expensive but also associated with health hazards. Proper processing of original RE ions is also very laborious and expensive, which are available only in limited geographical locations.^33,34^ In this context, RE-free materials delivering photo and persistent luminescence could be a great addition to a cost-effective phosphor library.

Here, we have explored the possibility of deploying the spinel template for invoking multifunctionality, lighting, and catalysis. We have probed pure spinels along with doped derivatives where Al^3+^ ions are incorporated at Ga^3+^ sites of the ZnGa_2_O_4_ spinel. The present work comprises synthesizing a series of samples starting from ZnGa_2_O_4_ to ZnAl_2_O_4_ by modulating Ga/Al ratio, and probing their luminescence and electrocatalytic properties. Since the octahedral B site is susceptible to easy modulation and leads to intriguing properties, it is expected to see similar changes in both luminescence and electrocatalytic studies. The synthesized materials were characterized by using powder X-ray diffraction (XRD), Fourier transform infrared spectroscopy (FTIR), and Raman spectroscopy. For determining the Ga/Al ratio, EDAX has been performed. In order to explore the emission as well as persistent luminescence characteristics with different Ga/Al ratios, photoluminescence (PL) was also carried out. The defect-related studies have been correlated using X-ray photoelectron spectroscopy (XPS), thermoluminescence (TL) and positron annihilation lifetime spectroscopy (PALS). Electrocatalytic activity, which includes both HER and OER of all materials, has also been performed.

## 2. Experimental

### 2.1. Synthesis

The spinel ZnGa_2−*x*_Al_*x*_O_4_ (*x* = 0 ≤ 2) were synthesized by conventional solid state reaction route. The precursors used are ZnO of SPEX Pure (99.999%), Al_2_O_3_ from Alfa Aesar (99.95%) and Ga_2_O_3_ of SPEX Pure (99.999%). Then in order to prepare the series of samples with different Ga/Al ratio the required amount of the precursors were weighed and grinded in a mortar and pestle for the complete mixing of precursors. Then it is calcined at 900 °C for 15 h with ramp temperature of 10 °C per minute in a tubular furnace. It was allowed to cool to room temperature followed by re-grinding. The second stage heating included sintering at 1200 °C for 15 h. The final grinding was carried out for 30 minutes to obtain the phase pure spinel compound. The schematic of the same has been shown in Fig. S1.†

### 2.2. Instrumentation

To evaluate the phase purity of the synthesized material XRD has been carried out by using a benchtop proto X-ray diffractometer equipped with the monochromatic X-ray source as Cu Kα (1.5405 Å). The XRD pattern recorded with a 2*θ* range from 15 to 80° with a scan rate of 2° min^−1^. All the measurements were done at an accelerating voltage of 30 kV and tube current at 20 mA. FTIR spectra were recorded on a Bruker Alpha FTIR spectrometer in pellet mode by making the pellet with KBr crystals. Raman spectral studies are carried out using a micro-Raman spectrometer (STR-300, SEKI Technotron, Japan). A 532 nm CW diode pumped solid state laser (DPSS, gem 532, laser quantum) is used as an excitation source. The spectrograph is calibrated using the 520.5 cm^−1^ line from silicon wafer. SEM-EDX analysis has done on a Tescan VEGA MV 2300 T SEM-EDS instrument. Photo physical studies have been carried out using Edinburg make fluorescence instrument of series FLS 1000 in which xenon lamp is the source. All thermoluminescence measurements are done using a commercial Risø TL/OSL-DA-20 reader. The XPS measurements were carried out on a SPECS instrument with a PHOBIOS 100/150 delay line detector (DLD). PALS measurements were carried out under ambient conditions where Na-22 encapsulated in 8 micron polyimide films was immersed in the powder sample. The resolution of the positron lifetime spectrometer was 185 ps. The spectra were analyzed using PALSFit software^35^ taking into account the resolution of the spectrometer and fraction of positrons annihilating in the polyimide films. Scanning Electron Microscopy (SEM) has been carried out on a SNE4500 Mini SEM instrument. All the ICP-AES analyses were carried out using a Spectro-Arcos SOP Unit, Ametek, Germany. The spectrometer is augmented with a detector set consisting of thermally stabilized linear arrays of CCD detectors (3648 pixels per array). High purity argon was used as the plasma gas, carrier gas and auxiliary gas as well. The non-LTE plasma was sustained with a constant RF supply of 40 MHz and 1.3 kW of forward power. To establish the standardization, four point calibration curves were established using 1, 10, 100 and 500 ppm of multi elemental standards which were prepared from the stock of Merck-IV ICP solution standard. All measurements were done in triplicate. High pure Ar gas (total flow rate of 15 liters per minute) was also used for flushing of the detector before all the measurements to minimize the background. All the electrochemical characterizations were performed using Metrohm Auto Lab PGSTAT 201 potentiostat with three electrode systems at room temperature (298 K). The pH of the electrolyte used was adjusted using ORION STAR A111 pH Meter (Thermo Scientific) or LMPH-9 (Labman Scientific). A spiral platinum electrode (Pt) and silver chloride electrode (Ag/AgCl in 3 M KCl) were taken as the counter and reference electrodes, respectively.

## 3. Results and discussions

### 3.1. Phase, vibrational spectroscopy and elemental analysis

**Fig. 1:**
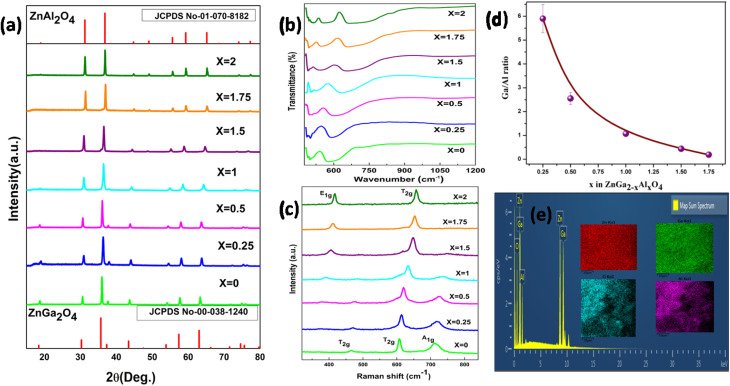
(a) Powder XRD pattern (b) FTIR spectra (c) Raman spectra of ZnGa_2−*x*_Al_*x*_O_4_ (*x* = 0, 0.25, 0.5, 1, 1.5, 1.75, 2) (d) Plot of Ga/Al ratio of ZnGa_2−*x*_Al_*x*_O_4_ (*x* = 0, 0.25, 0.5, 1, 1.5, 1.75, 2) and (e) element mapping results of O, Al, Ga, and Zn elements in the ZnGa_1_Al_1_O_4_.

Fig. 1a shows the XRD pattern of the synthesized samples. The pattern matches with the standard pattern of both ZnGa_2_O_4_ and ZnAl_2_O_4_ with JCPDS no. 00-038-1240 and 01-070-8182, respectively which implies the formation of phase pure material. There are no peaks of the starting precursors used. The major diffraction peaks of ZnGa_2_O_4_ are present at 2*θ* values of 18.7, 30.6, 36.0, 37.6, 43.7, 54.1, 57.7, 63.3, 71.8, 74.9 and 75.9 corresponding to (311), (220), (111), (222), (400), (422), (511), (440), (620), (533) and (622) crystal planes respectively. Likewise, for ZnAl_2_O_4_ the 2*θ* values are at 31.3, 36.9, 44.8, 49.1, 55.7, 59.4, 65.3, 74.1 and 77.3 corresponding to (220), (311), (400), (331), (422), (511), (440), (620) and (533) planes respectively. On increasing the Al ratio in the spinel, a shift in the diffraction peaks to higher angle is observed suggesting that there is a decrease in the *d*-spacing.

The FTIR spectrum is shown in Fig. 1b. In the spinel system the Al might coordinate to oxygen in two different ways, either it coordinates to 4 oxygen atoms (AlO_4_) forming a tetrahedral network or it can coordinate to 6 oxygen atoms (AlO_6_) forming an octahedron network. There are mainly 3 absorption peaks in the FTIR spectrum of ZnAl_2_O_4_; they are at 663, 554 and 515 cm^−1^ which represents the symmetric stretching vibration of Al–O bond, symmetric bending vibration of Al–O bond and asymmetric stretching vibration of Al–O bond respectively. Along with these peaks there is also a shoulder peak at 849 cm^−1^ which is due to the stretching vibration of Al–O bond in the AlO_4_ unit. This implies that some of the Al is occupying as AlO_4_ unit also,^36,37^ whereas in the FTIR spectrum of ZnGa_2_O_4_, there are mainly 2 peaks at 580 and 498 which represents the vibration of Ga–O–Zn and Zn–O bond.^38^ On moving from zinc gallate to zinc aluminate, evolution of new vibrational peak above 600 cm^−1^ is observed which represents the symmetric stretching of Al–O bond.

Fig. 1c represents the Raman spectra of synthesized samples [ZnGa_2−*x*_Al_*x*_O_4_ (*x* = 0 ≤ 2)]. According to group theory, ZnGa_2_O_4_ and ZnAl_2_O_4_ are expected to possess 5 Raman active modes (A_1g_ + E_g_ + 3T_2g_). These 5 modes are known as first order Raman active modes which are basically due to the motion of the Zn^2+^ in the tetrahedral site and not due to the motion of Ga^3+^ in the octahedral site. The Raman spectra of ZnGa_2_O_4_ show mainly three peaks at 466, 608 and 711 cm^−1^ which are two T_2g_ and A_1g_ modes respectively.^38^ The Raman spectra of ZnAl_2_O_4_ contains mainly 2 Raman peaks at 418 and 657 cm^−1^ which represents the E_1g_ and T_2g_ modes which correspond to asymmetric bending motion of oxygen in tetrahedral and octahedral site respectively. Due to the high intensity of E_1g_ and T_2g_ modes, other modes are not visible here.^39^ The intermittent compositions wherein *x* < 1 display the feature typical of ZnGa_2_O_4_ where as the one wherein *x* ≥ 1 are on line with spectra of ZnAl_2_O_4_.

The Ga/Al ratio has been calculated from the counts obtained in the EDAX spectra which have been given in Table 1 and the pictorial representation of the same can be seen in the Fig. 1d. In the elemental mapping results of one of the representative spinels ZnGa_1_Al_1_O_4_ (Fig. 1e), homogeneous distribution of all the elements could be clearly seen. Fig. S2† represents the EDAX mapping of all the other samples, which gives the information on elemental compositions. It shows the uniform distribution of elements throughout the sample. The elemental compositional analysis of the representative samples ZnGa_2_O_4_, ZnGa_1_Al_1_O_4_ and ZnAl_2_O_4_ has done by using ICP-AES. The procedure and the results are shown in ESI (Table S1).†

**Table 1:** EDAX based composition estimation of Ga/Al ratio in ZnGa_2−*x*_Al_*x*_O_4_ spinel

Sample	Atomic%	Ga/Al ratio
ZnGa_1.75_Al_0.25_O_4_	Ga – 20.92	5.9
	Al – 3.55	
ZnGa_1.5_Al_0.5_O_4_	Ga – 16.96	2.55
	Al – 6.66	
ZnGa_1_Al_1_O_4_	Ga – 13.63	1.07
	Al – 12.74	
ZnGa_0.5_Al_1.5_O_4_	Ga – 8.41	0.438
	Al – 19.22	
ZnGa_0.25_Al_1.75_O_4_	Ga – 4.21	0.19
	Al – 22.02	

### 3.2. Defect characterization: XPS and PALS

**Fig. 2:**
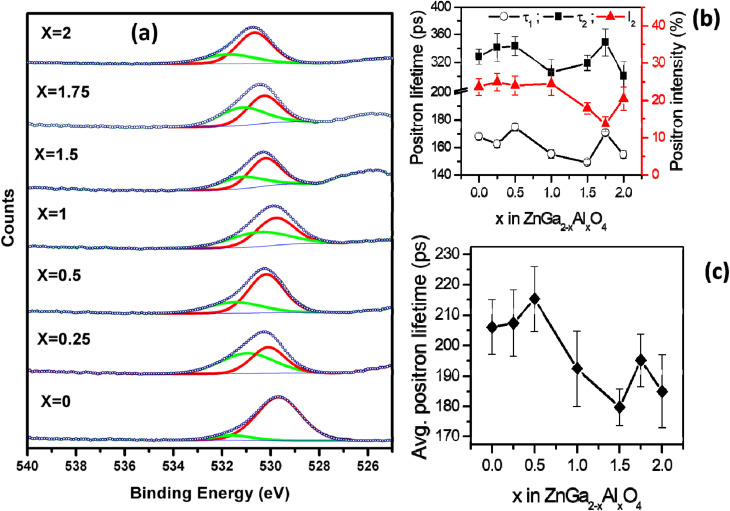
(a) O 1s core level XPS spectra of ZnGa_2−*x*_Al_*x*_O_4_. (*x* = 0, 0.25, 0.5, 1, 1.5, 1.75, 2) (b) positron lifetime data (c) average positron lifetime.

The survey scans of the representative samples (ZnGa_2_O_4_, ZnGa_1_Al_1_O_4_ and ZnAl_2_O_4_) are shown in Fig. S3† which confirms the surface composition of the materials. Fig. 2a represents the XPS core level spectra of oxygen which is deconvoluted into 2 peaks. The one with lower binding energy (∼529–530 eV, red curve) corresponds to the lattice oxygen and the other with higher binding energy (∼531 eV, green curve) corresponds to the oxygen vacancy.^40^ The atomic percentage of oxygen vacancies in different spinel phases are listed in Table 2. It can be seen that the highest concentration of oxygen vacancy is present in ZnGa_1.75_Al_0.25_O_4_. The core level XPS spectra of Zn, Al, Ga of the representative samples ZnGa_2_O_4_, ZnGa_1_Al_1_O_4_ and ZnAl_2_O_4_ are also shown in Fig. S4.† The two characteristic peaks of Zn 2p are located at 1021.8 and 1044.8 eV which corresponds to a peak spacing of 23 eV that is characteristic for divalent Zn ion (Zn^2+^). In Ga 2p XPS spectra the two characteristic peaks are located at 1115.8 and 1142.7 eV with peak spacing of 26.9 eV which corresponds to trivalent gallium ion (Ga^3+^).^41^ For Al 2p there is only one XPS peak at ∼74 eV which represents the Al^3+^ in AlO_6_ octahedra.^42^

**Table 2:** Atomic% of oxygen vacancy in ZnGa_2−*x*_Al_*x*_O_4_ spinel

Sample	Peak 2	Atomic% of oxygen vacancy
ZnGa_2_O_4_	531.6	8
ZnGa_1.75_Al_0.25_O_4_	531.0	55
ZnGa_1.5_Al_0.5_O_4_	531.4	30
ZnGa_1_Al_1_O_4_	531.1	45
ZnGa_0.5_Al_1.5_O_4_	531.1	36
ZnGa_0.25_Al_1.75_O_4_	531.2	47
ZnAl_2_O_4_	531.6	30

All the positron annihilation lifetime spectra could be fitted to sum of three exponentials. The three lifetimes obtained are numbered in the increasing order of the magnitude and the intensities corresponding to these lifetimes are referred to as *I*_1_, *I*_2_ and *I*_3_, as is the convention. The longest-lived component of lifetime ∼ 1 ns and intensity (*I*_3_) ∼ 0.6% was from positronium formation on the surface of the powder particles. Though this component was too small, inclusion of it in the fitting was necessary to obtain the best fit to the experimental spectra. The other two lifetimes (*τ*_1_ and *τ*_2_), their corresponding intensities (*I*_1_, *I*_2_ with *I*_1_ + *I*_2_ ∼ constant) and the intensity weighted average positron lifetime in these samples is given in the Fig. 2b and c. The uncertainities on the values of the individual lifetimes and intensities is from the fitting of the spectra while uncertainity on the average lifetime is calculated from the uncertainities of individual lifetimes and intensities used in calculating the average.

The first positron lifetime is in the range of 150–170 ps and the second positron lifetime is in the range of 300–350 ps. The first positron lifetime is from the positron annihilations in the bulk with contribution from shallow positron traps while the second component is from the vacancy clusters. It is also noticed that in the solid solutions, the variation in the positron lifetimes is not monotonous. The positron lifetimes are higher in ZnGa_2_O_4_ than ZnAl_2_O_4_. The intensity of the second lifetime is lower in Al rich samples when compared to Ga rich sample. The same is true of average positron lifetimes. The sample with *x* = 1 shows nearly weighted average positron lifetime of in ZnGa_2_O_4_ than ZnAl_2_O_4_ where as others show deviations from this weighted average.

### 3.3. Optical measurements

**Fig. 3:**
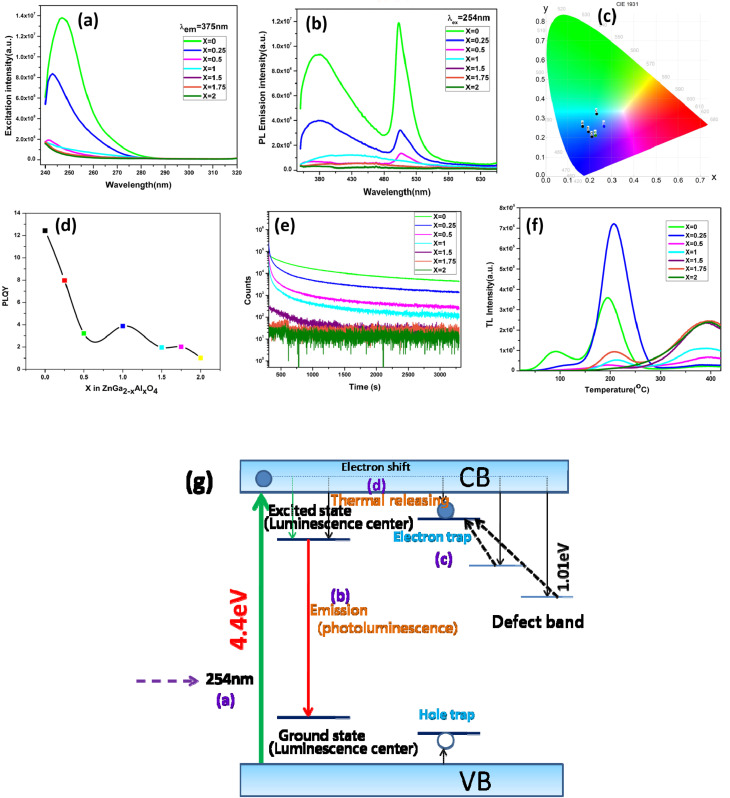
(a) Excitation spectra (b) emission spectra (c) CIE diagram (d) plot of quantum yield against concentration (e) persistent lifetime spectra (f) thermoluminescence spectra of ZnGa_2−*x*_Al_*x*_O_4_ (*x* = 0, 0.25, 0.5, 1, 1.5, 1.75, 2) (g) mechanism of the persistent luminescence.

Fig. 3a represents the excitation spectra of ZnGa_2−*x*_Al_*x*_O_4_ with a constant emission wavelength of 375 nm. It consists of a band near 254 nm which is due to the O^2−^ → Ga^3+^/Al^3+^/Zn^2+^ charge transfer. Fig. 3b is the emission spectra which is excited at 254 nm. There are mainly two bands observed in the emission spectra which are in the blue and green region located at 400 and 500 nm respectively. Normally ZnGa_2_O_4_ reported only have intrinsic blue emission due to the self activation of octahedral GaO_6_ unit.^43,44^ Appearance of bright blue PL around 400–460 nm in ZnGa_2_O_4_ has been reported earlier as well and it is indeed ascribed to the self-activation of GaO_6_.^45–47^ ZnGa_2_O_4_ nanocrystals PL peak with respect to its bulk counterpart is fairly narrower and blue-shifted by 35 nm.^48^

To tune the emission range in visible region, the normal way is to do the doping with different rare earth elements. But here the ZnGa_2_O_4_ produced *via* solid-state reaction route is showing emission in green region in addition to the blue region. This is due to the formation of vacancies at higher annealing temperature which subsequently create different defect energy levels between the two bands, thus making the visible range emission possible. The green emission is due to the presence of oxygen vacancies.^49^ Noto *et al.*^50^ have also reported similar kind of emission in ZnGa_2_O_4_ prepared by microwave assisted solid state reaction in the range of 380 to 660 nm and 665–740 nm. Till date we couldn't find any report on afterglow PL from undoped ZnGa_2_O_4_.

From the CIE diagram which is shown in Fig. 3c it is clear that although the material has emission peaks in blue, green and red region, the overall emission profile occurs in the blue region. With different Al content there is color tunability in the blue region. The corresponding *x*, *y* coordinates are shown in the Table S2.† On increasing the Al content and gradually replacing Ga^3+^ ion in ZnGa_2_O_4_ the photoluminescence quantum yield (PLQY) is getting reduced, the same trend is also observed in the emission intensity. ZnGa_2_O_4_ is having a higher quantum yield of ∼13% as can be seen from Fig. 3d.

In order to study the afterglow decay processes, the curve of all the samples is shown in the Fig. 3e. The samples have been irradiated by 254 nm UV lamp for 5 min. It is clear from the figure that ZnGa_2_O_4_ is showing a persistent luminescence of up to 55 minutes with appreciable counts. All the other samples are showing less persistent luminescence time. The presence of shallow traps in ZnGa_2_O_4_ is responsible for the persistent luminescence. With increase in the Al content the persistent luminescence has got degraded. From the decay curves it seen that the decay is a bi-exponential decay having a fast decaying and slow decaying component. The mechanism for the persistent luminescence is discussed in the later part of this section.

Fig. 3f shows the thermoluminescence (TSL) spectra of ZnGa_2−*x*_Al_*x*_O_4_ which is irradiated at 254 nm for 5 minutes then followed by heating the sample at a heating rate of 2 °C s^−1^. The experiment has been performed without using the filter. Shape of the glow curve determines the order of the kinetics. More precisely the term is defined as geometric shape factor or symmetry factor (*μ*_g_) which is given by

1 *μ*_g_ = *δ*/*ω*

where *δ* = *T*_2_ − *T*_M_ and *ω* = *T*_2_ − *T*_1_. *T*_M_ represents the peak temperature at the maximum and *T*_1_, *T*_2_ are temperatures on either side of the *T*_M_ corresponding to half intensity.^51^

In the figure ZnGa_2_O_4_ is having two different glow peaks which indicate that there are two types of traps present. Since the low temperature peak is overlapping with the higher temperature peak we are only calculating the trap parameters for the high temperature peak. The *T*_M_, *T*_1_ and *T*_2_ of the high temperature peak of ZnGa_2_O_4_ are 195.7 °C, 167 °C and 225.3 °C respectively. Substituting the values to the above equation gives the value of *μ*_g_ as 0.507, which suggest that the peak is obeying the second order kinetics, since the *μ*_g_ value for a first order and second order kinetics have a value of 0.42 and 0.52 respectively.^52^

According to Chen's method, which is independent of the order of the kinetics, the trap parameters can be calculated by using the eqn (2).^51^2
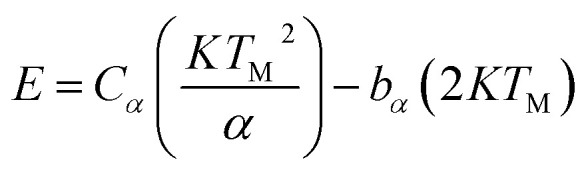


The corresponding activation energy is obtained as 1.013 eV. The frequency factor for a second order reaction has calculated by using the eqn (3).3
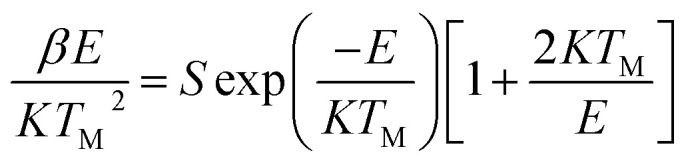
where *β* is the heating rate and *S* is the frequency factor. The frequency factor for ZnGa_2_O_4_ is 7.13 × 10^9^ s^−1^.

As it can be seen from the TSL data of the ZnGa_2_O_4_ sample, with relatively shallow traps has most favorable conditions to be used as a persistent luminescent material. This is evidenced from the persistent luminescence study also. With increase in Al content in the samples a small shift in the TL glow peak towards higher *T*_M_ was observed which is attributed to the increase in the activation energy related to deeper traps. From the figure it is also clear that the TSL intensity of ZnGa_1.75_Al_0.25_O_4_ is high compared to other materials which suggest that the concentration of traps formed is higher in this. From the persistent luminescence and TSL study a possible mechanism has been proposed which has been shown in Fig. 3g. Initially upon irradiation with a UV light large number of electrons and holes are formed which is represented as process (a). Then the energy is transferred to the luminescence centre through the lattice thus producing the rapid luminescence which is the process (b). And for the delayed luminescence the two defect centres are responsible. In the process (c) the carriers are get trapped into the defect centres. After thermal treatment the carriers are get detrapped and producing the persistent luminescence by the process (d).^53,54^

### 3.4. Electro catalytic study

#### 3.4.1. Electrocatalytic HER performance

**Fig. 4:**
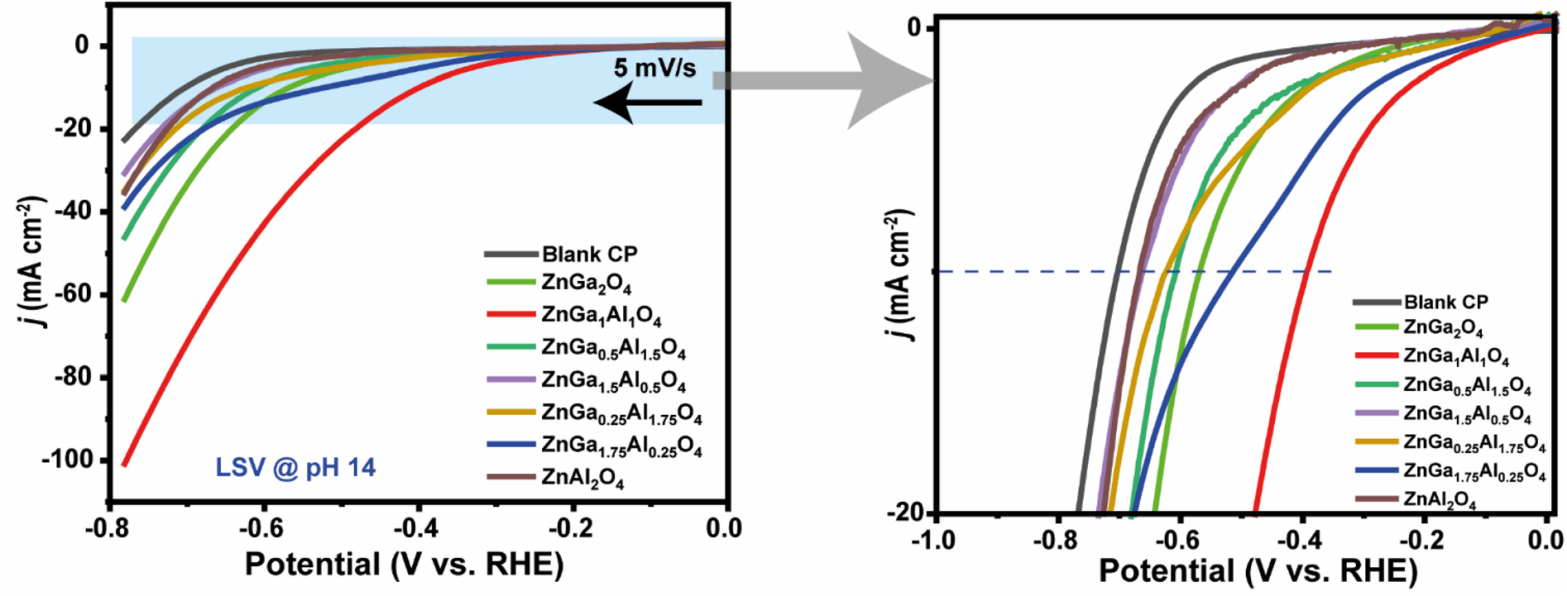
The left figure highlights the linear sweep voltammetry plots (HER) recorded for all the catalysts probed in this study in 1.0 M KOH solution (scan rate 5 mV s^−1^). The horizontal black arrow indicates the initial scan direction. The right hand side picture depicts the onset potential region for the HER activity.

A standard three-electrodes cell was used to perform both the HER and OER in an alkaline medium (1.0 M KOH) of pH 14.0. Fig. 4 shows the LSV curves (HER) for all synthesized catalysts. ZnGa_1_Al_1_O_4_ exhibited higher HER activity compared to the other compositions with a minimal overpotential of 390 mV to achieve a current density of 10 mA cm^−2^ (*j*_10_), which is significantly lower than ZnGa_2_O_4_ (570 mV/*j*_10_). The other electrocatalysts, ZnGa_1.75_Al_1.25_O_4_, ZnGa_0.5_Al_1.5_O_4_, ZnGa_0.25_Al_1.75_O_4_, ZnGa_1.5_Al_0.5_O_4_, ZnAl_2_O_4_, also exhibited high overpotential values of 515 mV, 610 mV, 622 mV, 659 mV and 667 mV to achieve 10 mA cm^−2^ current density (*j*_10_). When both the Al & Ga were present at an equimolar ratio (1 : 1) in the spinel, it exhibited the most efficient HER activity. The corresponding Tafel plot is shown in Fig. S5† where we have observed the lowest Tafel slope for ZnGa_1_Al_1_O_4_ sample. This data clearly indicates the fastest electrocatalytic HER kinetics for this ZnGa_1_Al_1_O_4_ material.

#### 3.4.2. Electrocatalytic OER performance

**Fig. 5:**
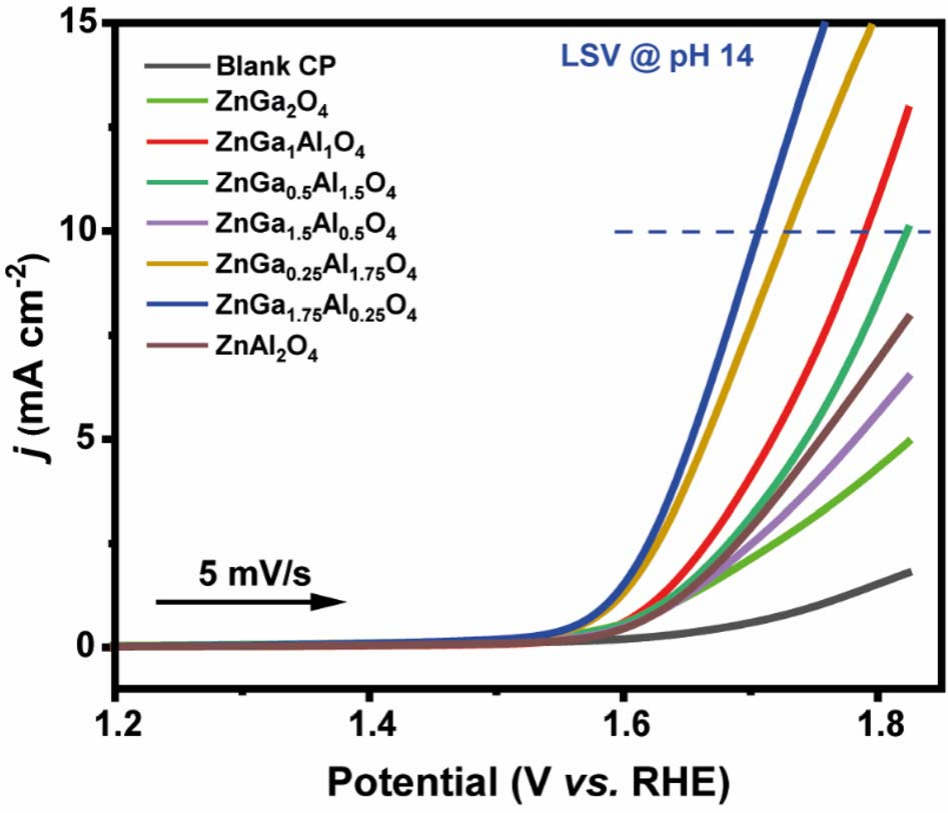
Linear sweep voltammetry plots (OER) recorded for all the catalysts probed in this study in 1.0 M KOH solution (scan rate 5 mV s^−1^). The horizontal black arrow indicates the initial scan direction.

The OER properties of the prepared catalysts were also probed in 1.0 M KOH electrolyte *via* LSV studies. Fig. 5 represents the LSV curves for OER activity for all the synthesized catalysts. ZnGa_1.75_Al_0.25_O_4_ demonstrated the best OER activity compared to the other catalysts, as it operates at 460 mV overpotential to achieve 10 mA cm^−2^ anodic current density (*j*_10_). On the other hand, ZnGa_0.25_Al_1.75_O_4_, ZnGa_1_Al_1_O_4_, and ZnGa_0.5_Al_1.5_O_4_ require 490 mV, 560 mV, and 590 mV of overpotential to achieve the same current density, respectively. The rest of the catalysts (ZnGa_2_O_4_, ZnGa_1.5_Al_0.5_O_4_, ZnAl_2_O_4_) exhibited poor OER response as all of them necessitate >800 mV of overpotential for significant O_2_ evolution under analogous conditions. The higher concentration of oxygen vacancies formed in ZnGa_1.75_Al_0.25_O_4_ which is obtained from XPS data is responsible for the higher OER activity. The change in concentration of oxygen vacancies with addition of Al can be explained in terms of antisite defects. Antisite disorder where cations are exchanged between tetrahedral and octahedral sites and Zn and oxygen vacancies are commonly reported defects in these materials.^55,56^ The antisite disorder is energetically more favorable in ZnGa_2_O_4_ than ZnAl_2_O_4_.^56^ Though the reasons are not clear at this juncture, the doping of Al in ZnGa_2_O_4_ which hinder antisite disorder might be causing more oxygen vacancies for charge compensation if Zn occupies octahedral sites.

Some of the previously reported electrocatalyst and their overpotential values are listed in Table S3.† It can be seen from this table that our OER and HER catalyst is having low overpotential values compared to those electrocatalysts. But we are aware of the fact that there are many more electrocatalysts with much lower overpotential values. So we would like to emphasize here that in this work, our aim is to unveil the structure–function relationship for Ga and Al-consisting Zn-spinels. Here, we have figured out the optimal ratio of Ga and Al for anodic OER and cathodic HER activity. Hence, we can now utilize different variants of the Zn-spinels in an electrolyser to drive the complete water-splitting.

#### 3.4.3. Electrochemical impedance spectroscopy

The electrochemical characterizations of all catalysts were further analyzed by Electrochemical Impedance Spectroscopy (EIS).^57^ The Nyquist plots were measured by the application of a sinusoidal wave with AC amplitude of 10 mV from 10^5^ Hz to 0.1 Hz operating frequency range. To understand the better activity of ZnGa_1.75_Al_0.25_O_4_ catalyst towards OER, EIS was measured with DC applied potential 1.55 V, 1.6 V, and 1.65 V w.r.t RHE. Here we have compared the charge transfer resistance of all catalysts at 1.6 V applied potential.

**Fig. 6:**
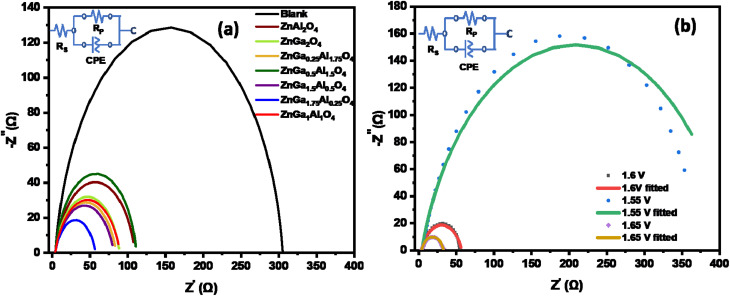
Nyquist plot of (a) all synthesized catalysts at 1.6 V *vs.* RHE in 1.0 M KOH solution and (b) ZnGa_1.75_Al_0.25_O_4_ catalyst.

All data are fitted by using an *R*(RC) circuit. The total electrical equivalent model consists of a solution resistance (*R*_S_), in a series with a parallel connection of internal resistance (*R*_P_) and a double-layer capacitance (*C*_dl_). *R*_P_ = *R*_S_ + *R*_ct_ can be illustrated as the sum of bulk electrolyte resistance and charge transfer resistance. For solid electrodes, double-layer capacitance can be replaced by a constant phase element (CPE).^57^ The fitted data based on Fig. 6a are summarized in Table 3. The less *R*_ct_ (47.3 ohms) of ZnGa_1.75_Al_0.25_O_4_ indicates better electro-catalytic activity for OER compared to the other synthesized catalysts at the same applied potential (1.6 V). Fig. 6b shows the Nyquist plot of ZnGa_1.75_Al_0.25_O_4_ at 1.55 V, 1.6 V, and 1.65 V *vs.* RHE. Dotted lines are original data and solid lines are fitted one. All the data are fitted by using an *R*(RC) circuit with a minimal error (*χ*^2^, 0.05 to 0.08).

**Table 3:** EIS fitted data from the Nyquist plot

Electrodes	*R*_S_ (ohm)	*R*_P_ (ohm)	*R*_CT_ (ohm)
Blank carbon paper	4.1	303.19	299.1
ZnGa_2_O_4_	4.12	84.97	80.85
ZnAl_2_O_4_	4.08	106	101.92
ZnGa_1.5_Al_0.5_O_4_	4.16	79.5	75.34
ZnGa_0.5_Al_1.5_O_4_	4.02	105.51	101.49
ZnGa_1_Al_1_O_4_	4.06	84.29	80.23
ZnGa_0.25_Al_1.75_O_4_	4.3	80.7	76.4
ZnGa_1.75_Al_0.25_O_4_	3.97	51.2	47.23

#### 3.4.4. Durability test

**Fig. 7:**
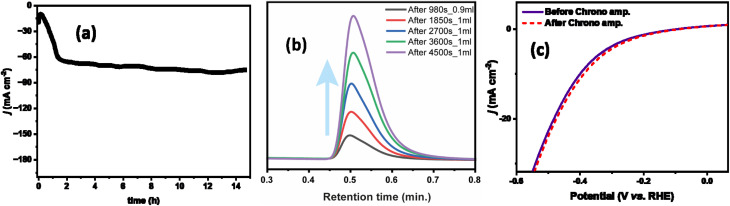
(a) Time dependence of current density of ZnGa_1_Al_1_O_4_ catalyst under a constant potential of −1.45 V, (b) volume of hydrogen gas measured during chronoamperometry test by gas chromatography, and (c) polarization curves, before and after chronoamperometry test of ZnGa_1_Al_1_O_4_ catalyst.

Besides the electrocatalytic performance, the durability test is one of the major parameters for understanding large industrial-scale applications. Polarization curves confirmed that ZnGa_1_Al_1_O_4_ showed less overpotential towards alkaline hydrogen evolution reaction among all other synthesized catalysts. Therefore, to measure the HER stability of the ZnGa_1_Al_1_O_4_ catalyst, chronoamperometric tests were assessed under a constant potential of −1.45 V (Fig. 7a). The cathodic current initially increased with time and then got stabilized at −76.5 mA cm^−2^ which indicates the good stability behavior of the catalyst.

The current density was slightly changed after 15 hours of chronoamperometry due to the sluggish mass transfer of HER and the blockage of some active sites of the catalyst. However, this catalyst exhibited fascinating faradaic efficiency of 88.7%. During the chronoamperometry test, the volume of H_2_ increased concerning time (Fig. 7b).

As shown in Fig. 7c, before and after the stability test the ZnGa_1_Al_1_O_4_ catalyst exhibited almost the same polarization curve with a slightly smaller overpotential of 360 mV to attain *j*_10_. Also we have varied the current density from 50 mA cm^−2^ to 250 mA cm^−2^, and observed a regular change in the potential. This experiment was performed over a period of ∼16 hours, where the potential remains stable at a current density of 250 mA cm^−2^. This has shown in Fig. S6.† The leaching of the aluminum is not observed in the electrolyte and it is below the detection limit of the instrument (<1 ppm). This suggests the good stability of the electrocatalysts. ICP results are shown in Table S4.†

## 4. Conclusion

The present work comprises the synthesis of solvent-free one pot synthesis of ZnGa_2_O_4_ to ZnAl_2_O_4_ by changing the Ga/Al ratio *via* solid state reaction. The materials have been characterized by using XRD, FTIR, Raman, XPS, PALS and EDAX. ZnGa_2_O_4_ showed a persistent luminescence of about 60 min with appreciable counts and the mechanism for the same has been proposed by using thermoluminescence studies. Al^3+^ incorporation reduces both PL and persistent luminescence duration owing to reduction in shallow traps and increase in non-radiative channels. Optical characterization indicates that ZnGa_2_O_4_ spinel displays excellent PL properties, with PLQY ∼ 13%. Moreover, due to synergistic effect of Al^3+^ incorporation and formation of oxygen vacancy, the resulting solid-state solution of ZnGa_2_O_4_ and ZnAl_2_O_4_ catalyst showed a bifunctional catalytic activity and superior stability for the OER and HER reaction with very low charge transfer resistance for the former and high faradaic efficiency of ∼90% for the later. Apart from the use of ZnGa_1_Al_1_O_4_ as electrocatalysts for HER, ZnGa_1.75_Al_0.25_O_4_ for OER catalysts, the unique PL and afterglow properties of ZnGa_2_O_4_ indicate a promising potential of these materials for energy and solid-state lighting applications.

## Conflicts of interest

There are no conflicts of interest to declare.

## Supplementary Material

RA-013-D3RA05017C-s001
