# Supplementary material for: ZnGa2−xAlxO4 (x = 0 ≤ 2) spinel for persistent light emission and HER/OER bi-functional catalysis
Source: RSC Adv. 2023 Oct 24;13(44):31101–11. doi: 10.1039/d3ra05017c (PMC10594079; doi:10.1039/d3ra05017c)
Supplement: RA-013-D3RA05017C-s001 [file RA-013-D3RA05017C-s001.pdf]

Electronic Supplementary Material (ESI) for RSC Advances.
This journal is © The Royal Society of Chemistry 2023

$ZnGa_{2-x}Al_xO_4$ (x=0≤2) Spinel for Persistent Light Emission and HER/OER Bi-functional Catalysis

Reshmi Thekke Parayil [1,2], Santosh K. Gupta,[1,2*] Manodip Pal,[3] Arnab Dutta,[3,4#] Deepak Tyagi,[5] Kathi Sudarshan,[1,2] Manoj Mohapatra,[1,2]

[1]Radiochemistry Division, Bhabha Atomic Research Centre, Trombay, Mumbai-400085, India
[2]Homi Bhabha National Institute, Anushaktinagar, Mumbai – 400094, India
[3]Chemistry Department, Indian Institute of Technology Bombay, Powai, Mumbai, 400076 India
[4]Interdisciplinary Program in Climate Studies, Indian Institute of Technology Bombay, Powai, Mumbai, 400076 India
[5]Chemistry Division, Bhabha Atomic Research Centre, Trombay, Mumbai-400085, India

*To whom correspondence should be addressed. Electronic mail: *santoshg@barc.gov.in (SKG)/ #arnab.dutta@iitb.ac.in (AD)*

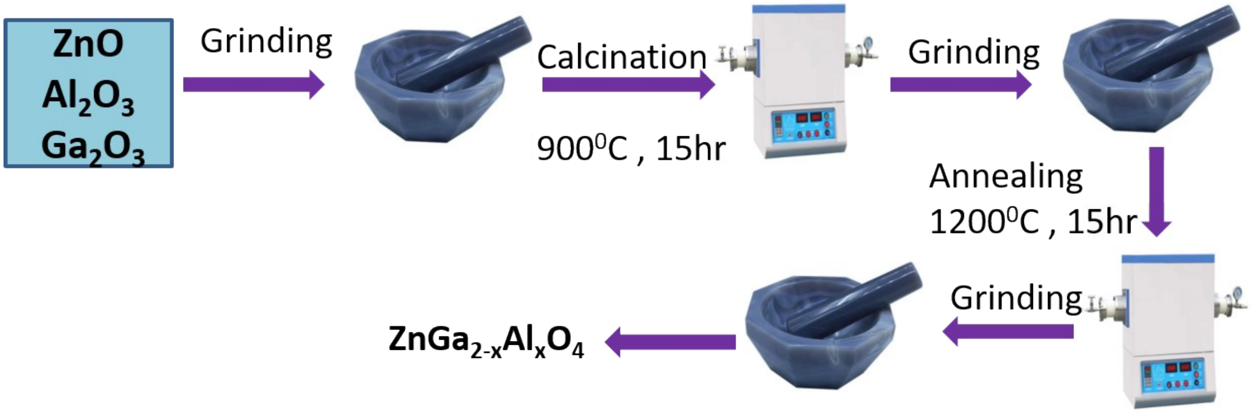

Figure S1: Schematics of the solid state synthesis of spinel $ZnGa_{2-x}Al_xO_4$ (x=0≤2)

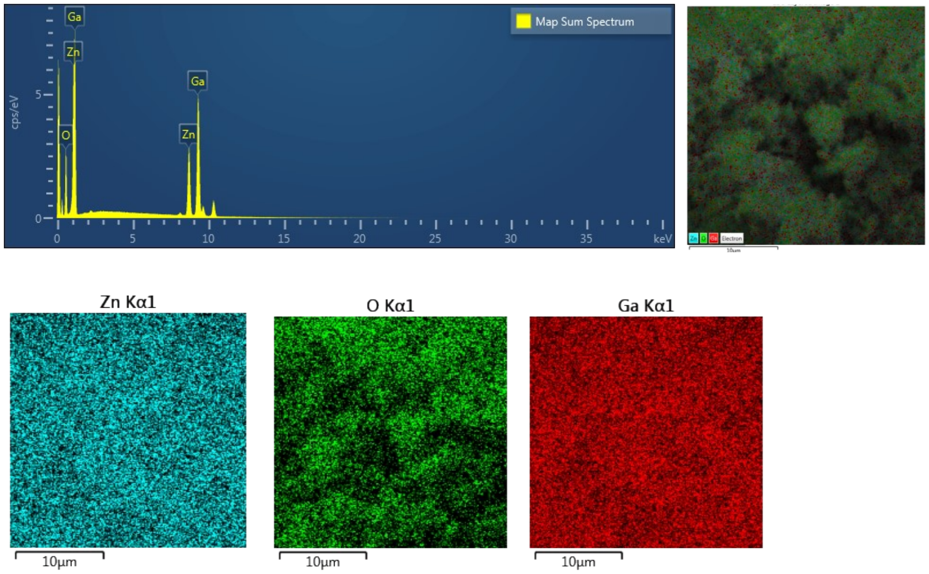

Figure S2a: EDAX spectra and elemental mapping of $ZnGa_2O_4$

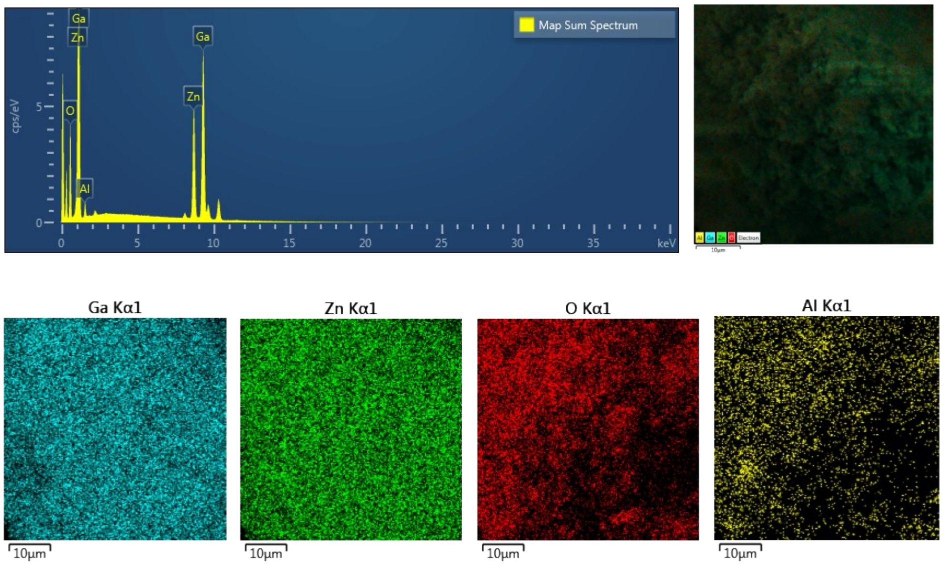

Figure S2b: EDAX spectra and elemental mapping of $ZnGa_{1.75}Al_{0.25}O_4$

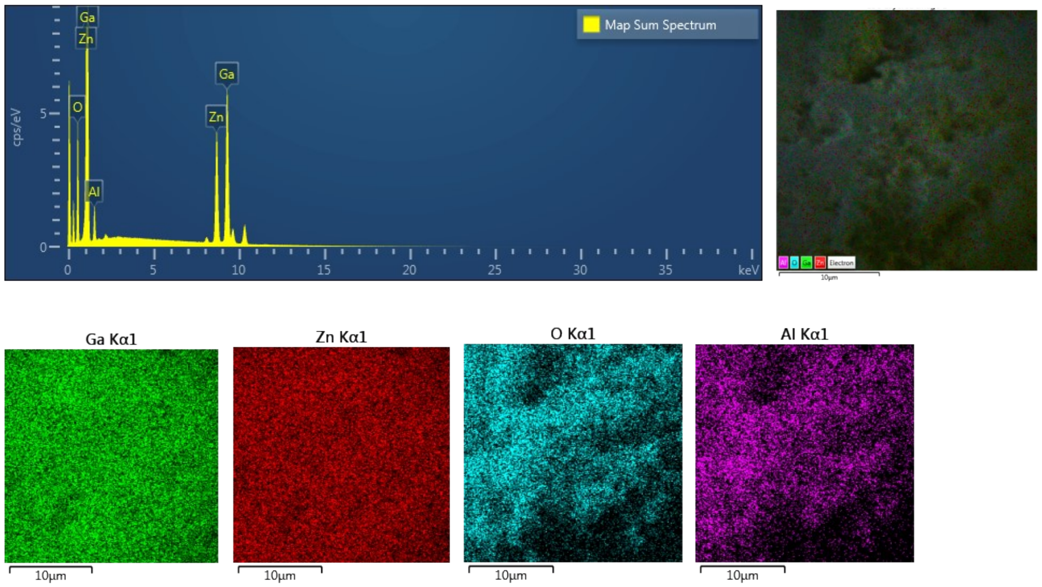

Figure S2c: EDAX spectra and elemental mapping of $ZnGa_{1.50}Al_{0.50}O_4$

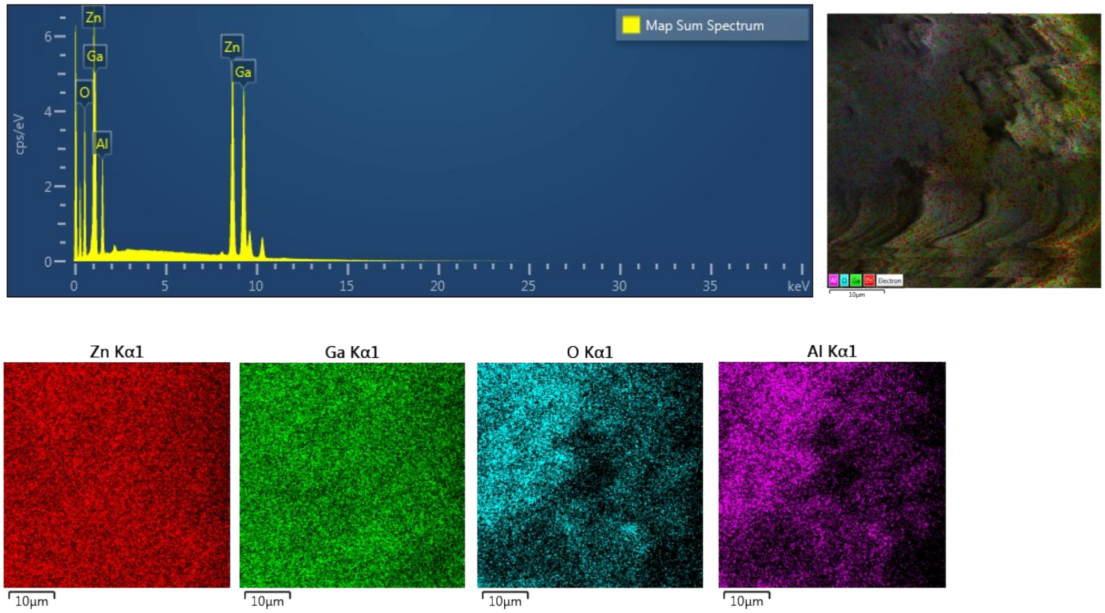

Figure S2d: EDAX spectra and elemental mapping of $ZnGa_1Al_1O_4$

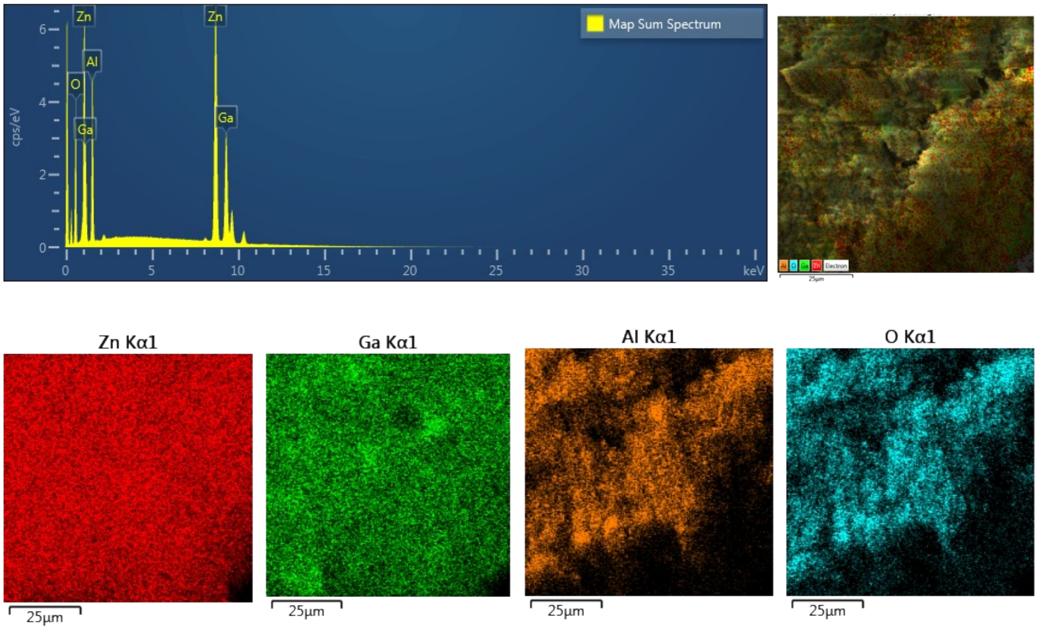

Figure S2e: EDAX spectra and elemental mapping of $ZnGa_{0.50}Al_{1.50}O_4$

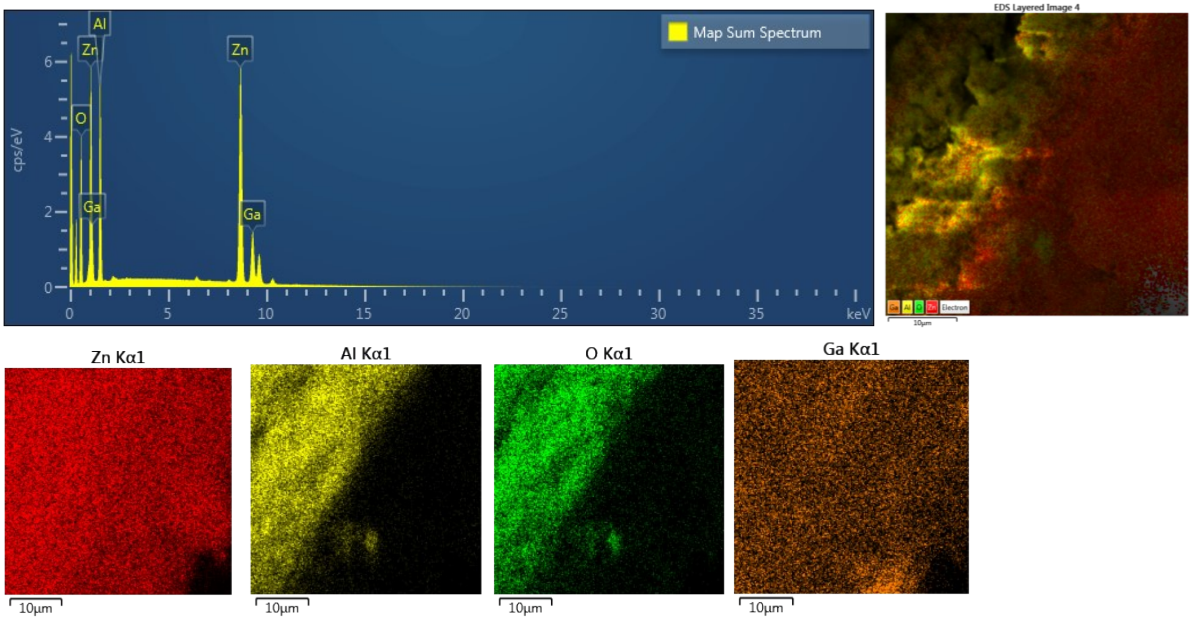

Figure S2f: EDAX spectra and elemental mapping of $ZnGa_{0.25}Al_{1.75}O_4$

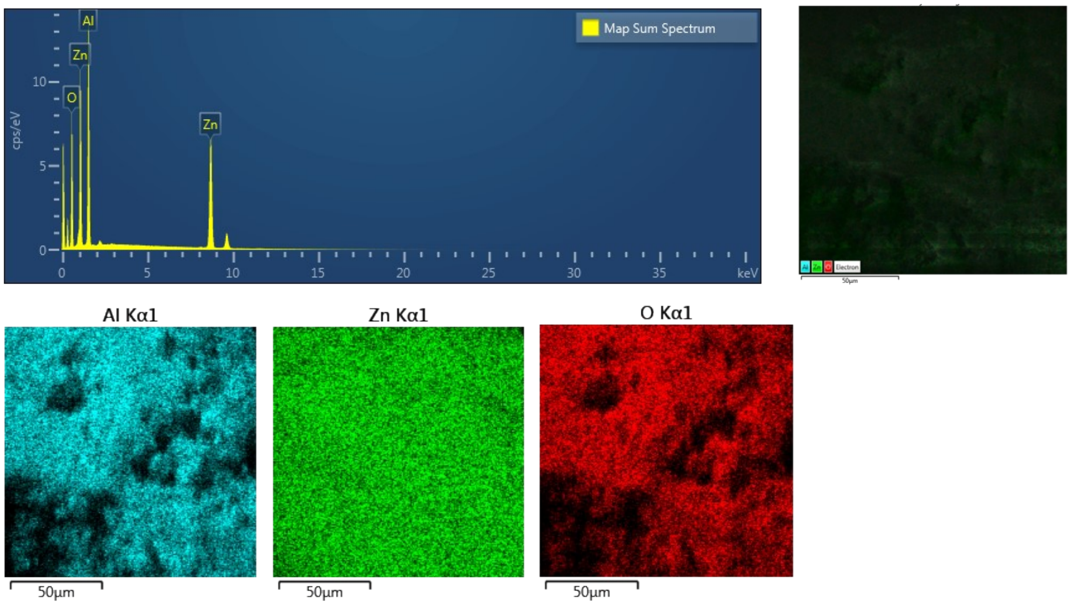

Figure S2g: EDAX spectra and elemental mapping of $ZnAl_2O_4$

<u>ICP-AES</u>

Since ICP-AES (Inductively Coupled Plasma- Atomic Emission Spectrometry)is primarily a trace analytical tool, in order to get a clear picture of the major matrix and the impurities present in the samples, a two step procedure was adopted. First, all the solid phosphor samples were digested in conc. HNO_3, the solutions were evaporated to near dryness. This procedure was repeated 3 times with lower acidities so as to get the final solution in 1M concentration. Each of these solutions was divided into two equal parts, one part was kept for the analysis of the major matrix and the other was for trace.

The solutions kept for the bulk analysis of Zn, Ga and Al, were diluted further so as to bring down the overall salt content to the tolerance levels of ICP instrument. Whereas, the solutions kept for the trace analyses were fed to the ICP directly without any dilution.

Table S1:ICP-AES results of the samples for the common metallic impurities content with a precision of ±10% RSD.

Elements	$ZnGa_2O_4$	$ZnGa_1Al_1O_4$	$ZnAl_2O_4$
Al	<1.0	1055	2060
B	<1.0	<1.0	<1.0
Be	<1.0	<1.0	<1.0
Ca	1.01	<1.0	<1.0
Cd	<1.0	<1.0	<1.0
Co	<1.0	<1.0	<1.0
Cr	<1.0	<1.0	<1.0
Cu	<1.0	<1.0	<1.0
Fe	<1.0	1.05	<1.0
Ga	2550	1038	1.2
Mg	<1.0	1.01	<1.0
Mn	<1.0	<1.0	<1.0
Mo	<1.0	<1.0	<1.0
Na	1.02	<1.0	1.05
Ni	<1.0	<1.0	<1.0
Pb	<1.0	<1.0	<1.0
Si	<1.0	<1.0	1.0
Sn	<1.0	<1.0	<1.0
V	<1.0	<1.0	<1.0
W	<1.0	<1.0	<1.0
Zn	1268	1075	1050

Table S2: Color coordinate values of $ZnGa_{2-x}Al_xO_4$ (x=0, 0.25, 0.5,1, 1.5, 1.75, 2)

Sample name	x	y
a- $ZnGa_2O_4$	0.16713	0.25722
b- $ZnGa_{1.75}Al_{0.25}O_4$	0.19355	0.23005
c- $ZnGa_{1.5}Al_{0.5}O_4$	0.23024	0.32176
d- $ZnGa_1Al_1O_4$	0.20991	0.20412
e- $ZnGa_{0.5}Al_{1.5}O_4$	0.22552	0.21038
f- $ZnGa_{0.25}Al_{1.75}O_4$	0.22517	0.20927

g- $ZnAl_2O_4$	0.26422	0.25748

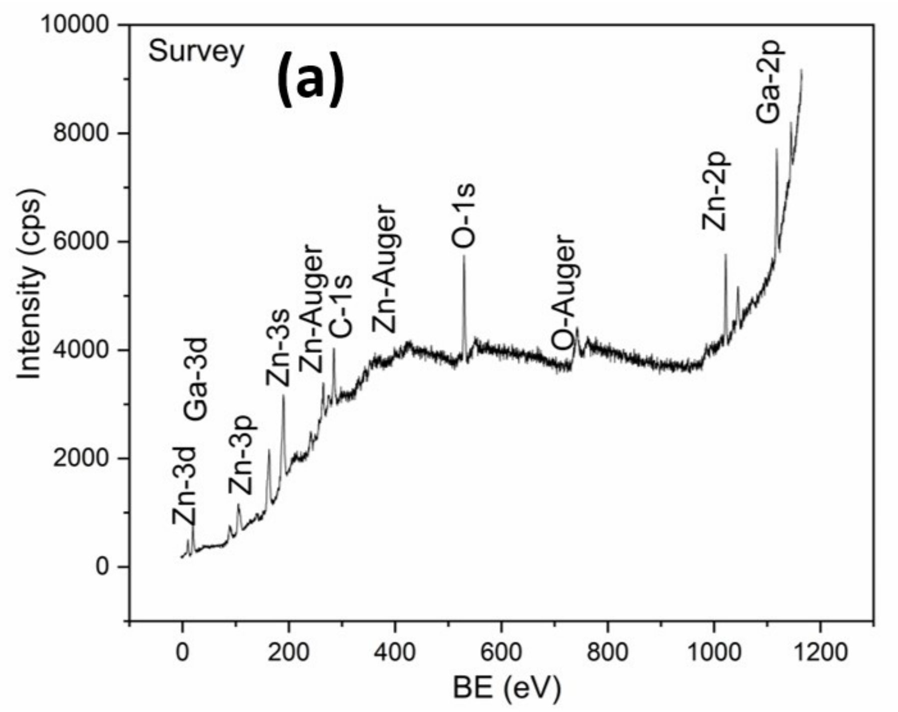

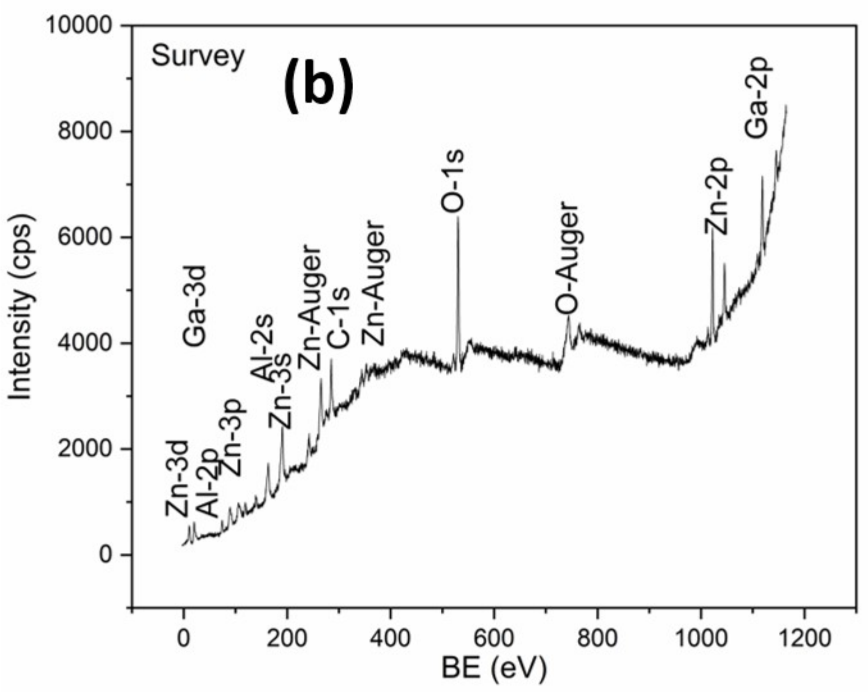

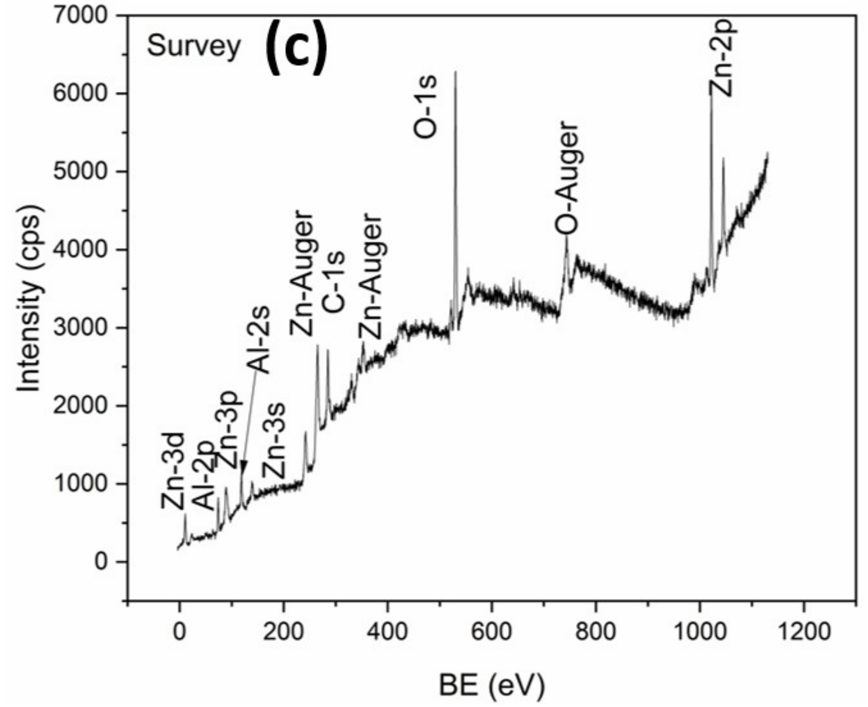

Figure S3: XPS survey scan spectra of (a) $ZnGa_2O_4$, (b)$ZnGa_1Al_1O_4$, and (c) $ZnAl_2O_4$

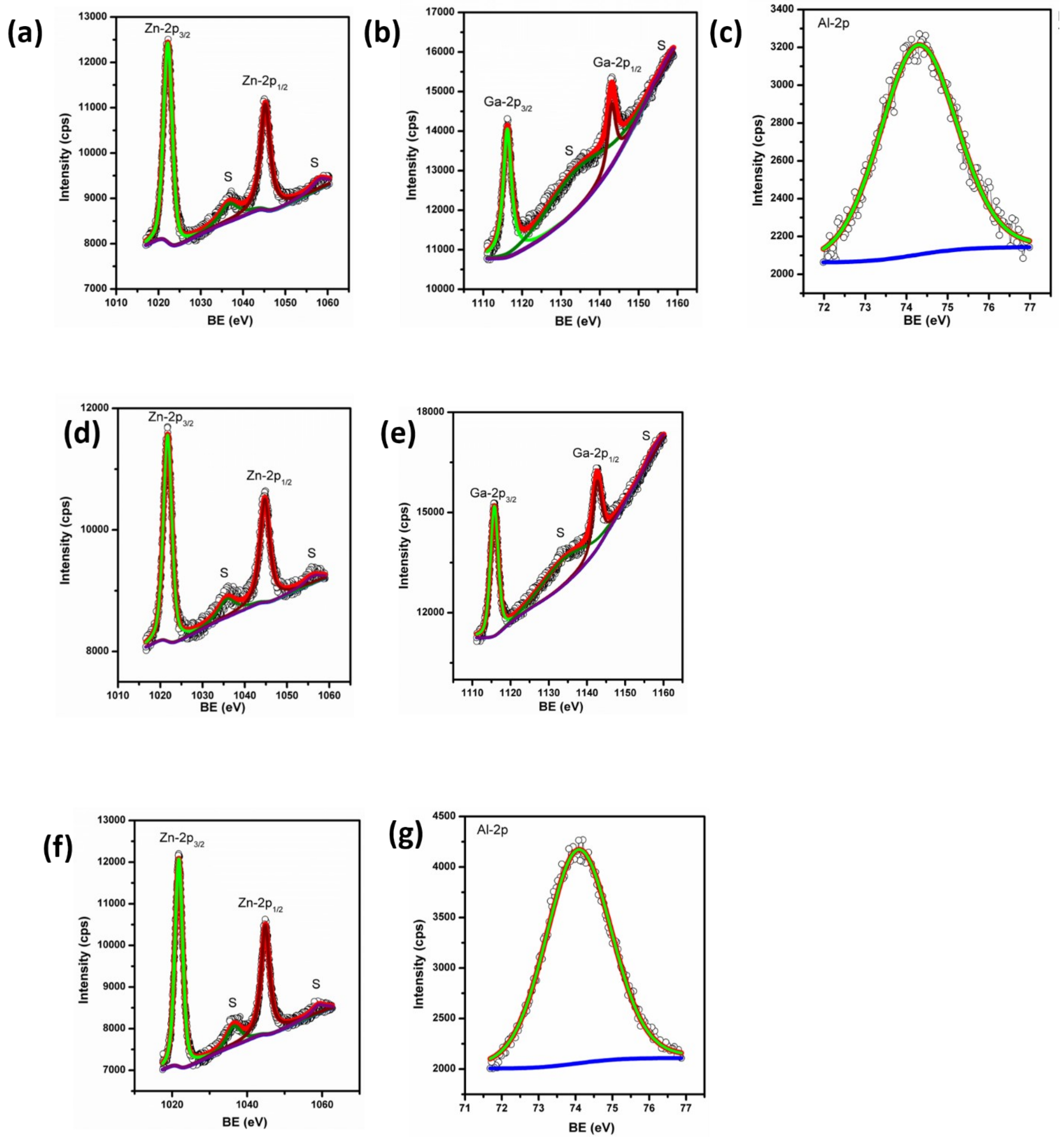

Figure S4: XPS core level spectra of (a-c) $ZnGa_1Al_1O_4$, (d-e) $ZnGa_2O_4$and (f-g) $ZnAl_2O_4$

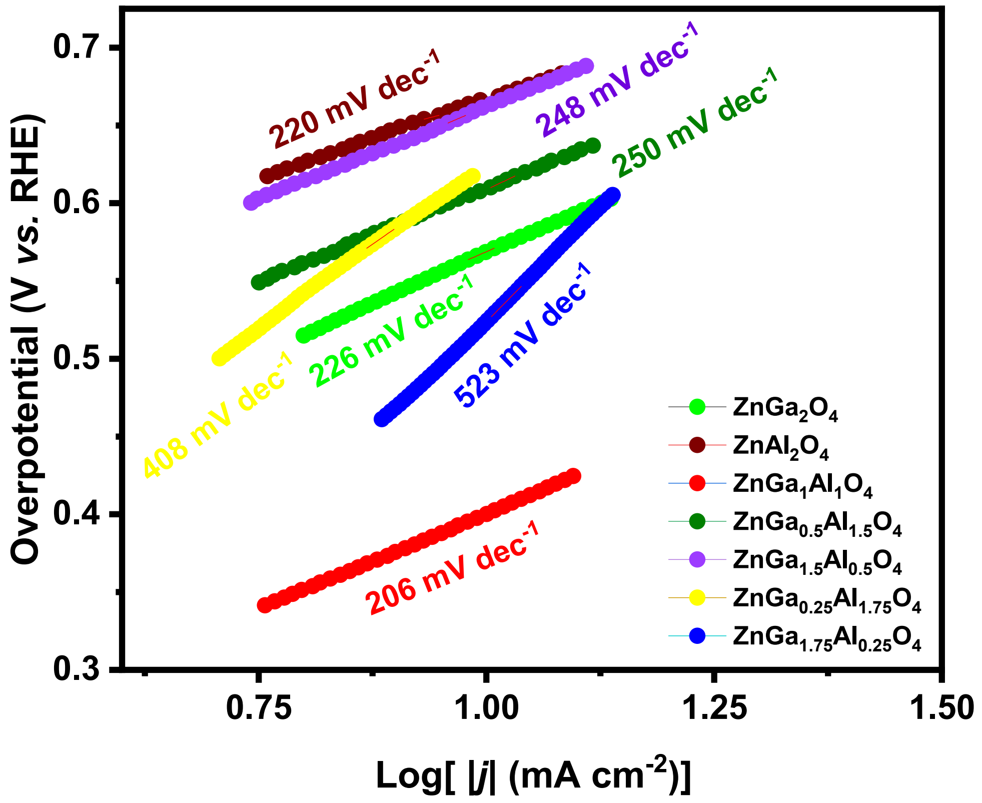

Figure S5: Tafel plot of the electrocatalysts

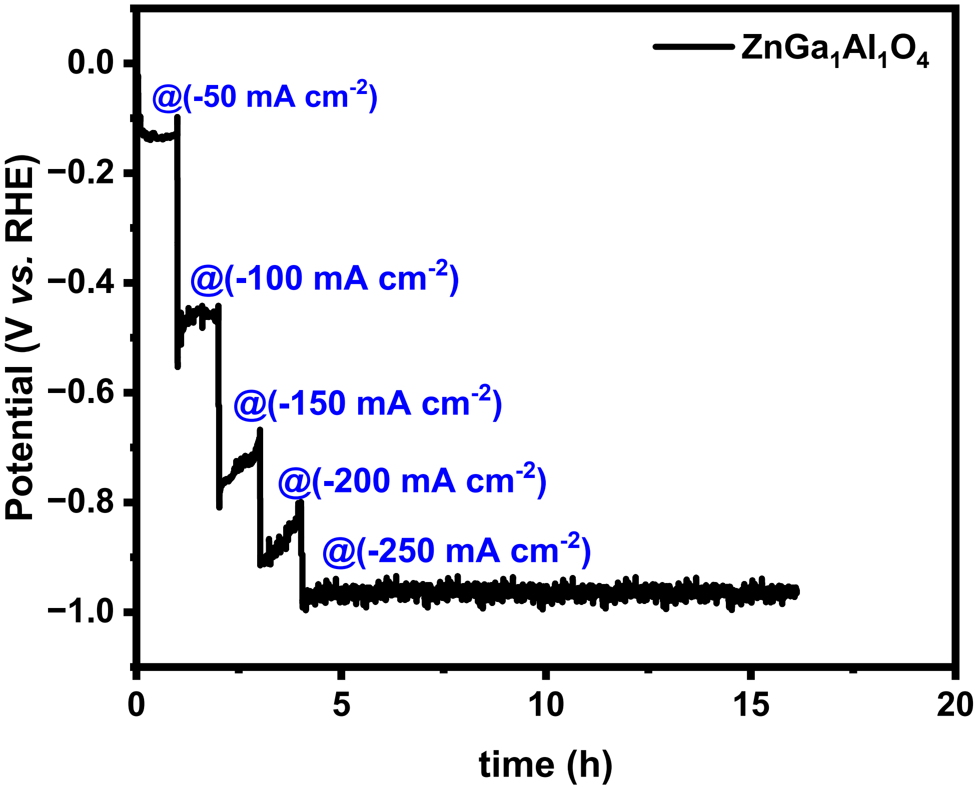

Figure S6: Multicurrent step test

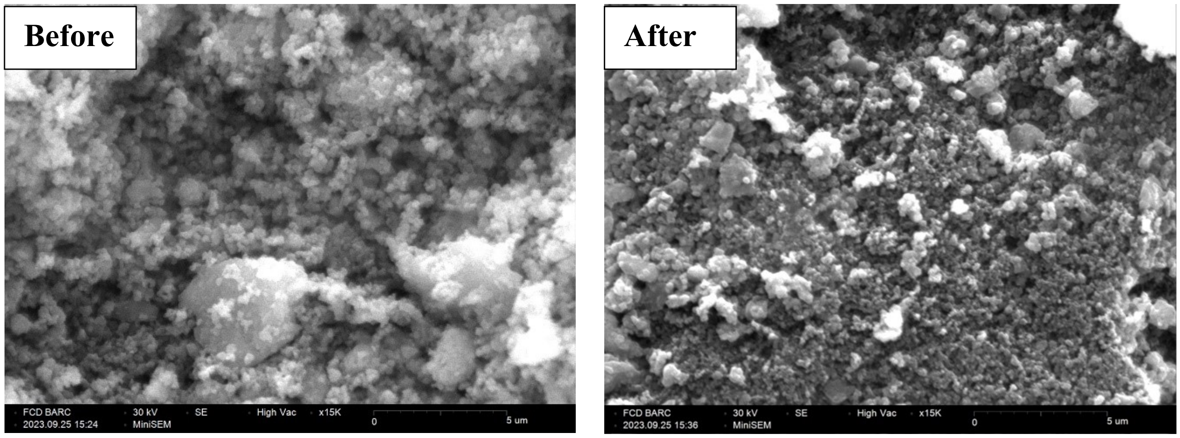

Figure S7: The SEM data for $ZnGa_1Al_1O_4$ material recorded before and after the electrocatalysis reaction.

Table S3: Overpotential values of some of the reported HER/OER electrocatalysts

Material	Overpotential (mV)	Electrolyte	HER/OER	References
Nano structured MnO_2	500	1M NaOH	OER	[1]
Co-Pi@CCH NA/Ti	460	0.1M Phosphate buffer	OER	[2]
Bulk $CoSe_2$	590	0.1M KOH	OER	[3]
Mn oxide	540	0.1M KOH	OER	[4]
Pristine $CoFe_2O_4$	470	1M KOH	OER	[5]
Ti_3C_2 Nanowire	476	0.5M H_2SO_4	HER	[6]
NiO hollow microspheres	424	1M KOH	HER	[7]
$ZnGa_1Al_1O_4$	390	1M KOH	HER	This work
$ZnGa_{1.75}Al_{0.25}O_4$	460	1M KOH	OER	This work

ICP-AES

The KOH electrolyte used for the OER/HER investigations were diluted with Type 2 water (Model Elix Essential, Merck Millipore, Germany) with a ratio of 1:100 in order to lower the pH and reduce the possible ionic interference from Potassium ions.

Table S4: ICP-AES results of the samples and the electrolyte for the common metallic impurities content with a precision of ±10% RSD.

Elements	KOH solution<br>After chronoamperometry
Al	<1.0
B	<1.0
Be	<1.0
Ca	<1.0
Cd	<1.0
Co	<1.0
Cr	<1.0
Cu	<1.0
Fe	<1.0
Ga	<1.0
Mg	<1.0
Mn	<1.0
Mo	<1.0
Na	<1.0
Ni	<1.0
Pb	<1.0
Si	<1.0
Sn	<1.0
V	<1.0
W	<1.0
Zn	<1.0

References:

[1] M. Fekete, R.K. Hocking, S.L.Y. Chang, C. Italiano, A.F. Patti, F. Arena, L. Spiccia, Energy & Environmental Science, 6 (2013) 2222-2232.

[2] L. Cui, D. Liu, S. Hao, F. Qu, G. Du, J. Liu, A.M. Asiri, X. Sun, Nanoscale, 9 (2017) 3752-3756.

[3] Y. Liu, H. Cheng, M. Lyu, S. Fan, Q. Liu, W. Zhang, Y. Zhi, C. Wang, C. Xiao, S. Wei, B. Ye, Y. Xie, Journal of the American Chemical Society, 136 (2014) 15670-15675.

[4] Y. Gorlin, T.F. Jaramillo, Journal of the American Chemical Society, 132 (2010) 13612-13614.

[5] J. Sun, N. Guo, Z. Shao, K. Huang, Y. Li, F. He, Q. Wang, Advanced Energy Materials, 8 (2018) 1800980.

[6] W. Zhao, B. Jin, L. Wang, C. Ding, M. Jiang, T. Chen, S. Bi, S. Liu, Q. Zhao, Chinese Chemical Letters, 33 (2022) 557-561.

[7] A. Mondal, A. Paul, D.N. Srivastava, A.B. Panda, International Journal of Hydrogen Energy, 43 (2018) 21665-21674.
